# *Chlamydia trachomatis* infection rates among a cohort of mobile soldiers stationed at Fort Bragg, North Carolina, 2005–2010

**DOI:** 10.1186/1471-2458-14-181

**Published:** 2014-02-20

**Authors:** Shilpa Hakre, Robert J Oyler, Kenneth A Ferrell, Fang Li, Nelson L Michael, Paul T Scott, Bruno P Petruccelli

**Affiliations:** 1 United States Military HIV Research Program, Henry M. Jackson Foundation for the Advancement of Military Medicine, Bethesda, MD, USA; 2 Womack Army Medical Center, Fort Bragg, NC, USA; 3 United States Military HIV Research Program, Walter Reed Army Institute of Research, Bethesda, MD, USA

**Keywords:** Chlamydia, Army, Mobility

## Abstract

**Background:**

Fort Bragg, a large Army installation with reported high *Chlamydia trachomatis* (Ct) infection rates, is characterized by a highly mobile population and a surrounding Ct-endemic community. We assessed the rates of Ct incidence and recurrence among the installation’s active component Army personnel and determined the association of soldier transience, sociodemographic factors, and history of sexually transmitted infection (STI) with these rates.

**Methods:**

A cohort of soldiers stationed at Fort Bragg during 2005 to mid-2010 was followed for incident and recurrent Ct infection using laboratory-confirmed reportable disease data. Linkage to demographic and administrative data permitted multivariate analysis to determine association of covariates with initial or recurrent infection.

**Results:**

Among 67,425 soldiers, 2,198 (3.3%) contracted an incident Ct infection (crude incidence, 21.7 per 1,000 person-years). Among soldiers followed for incident infection, 223 (10.6%, crude incidence 110.8 per 1,000 person-years) contracted a recurrent Ct infection. Being female, of lower rank, under 26 years of age, of non-white race, single, or with a high school diploma or less was significantly associated with incident Ct infection. Having breaks in duty or having deployments during follow-up was associated with a lower infection rate. Among women, having prior deployments was associated with a lower rate of both incident and recurrent infection. Specifically associated with recurrent infection in women was age under 21 years or no education beyond high school.

**Conclusions:**

This analysis reaffirms risk factors for Ct infection determined in other studies. In addition, infection risk was lower for more mobile soldiers and tied to the specific location of their regular duty assignment. The findings support the STI prevention efforts at Fort Bragg and the surrounding community, regardless of how often or for how long soldiers have deployed for military operations.

## Background

Historically, the targeting of military populations for sexually transmitted infection (STI) preventive interventions—including enhanced case contact tracing, condom distribution, and education campaigns—was associated with the deployment of troops for overseas combat duty [1,2]. However, the manner in which warfare is conducted, the prevailing cultures of regions where U.S. troops principally deploy, and the prevailing social dynamics that drive STI transmission regardless of war have all changed significantly since the major military conflicts of the 20^th^ century. Military populations tend to have periods of absence from an otherwise stable location, especially in wartime; and while this tendency can confound efforts to identify disease transmission foci, it also provides an opportunity for epidemiologists to weigh the mobility of the at-risk population against the frequency of disease emergence or detection at the locations where troops have their long-term duty assignment.

Many non-military populations are at least as mobile as the average soldier, airman, sailor or Marine. A study by Goldenberg and colleagues suggests that the transience of young oil and gas workers spending time in one small British Columbia city exacerbated promiscuity in the local population [3]. Koizumi and colleagues used mapping with spatial analysis to reveal an ecological correlation between the presence of military bases in specific Virginia counties, and county-level rates of *Chlamydia trachomatis* (Ct) and gonorrhea (GC); but the area studied is in a part of the tidewater region where population-dense foci might account for higher disease rates without attribution to military subpopulations [4]. While these studies indirectly account for non-permanent residents of given geographic locations, analyses that specifically address the mobility of subpopulations are generally lacking.

Few studies have addressed STI rates and risk factors in U.S. military personnel as they relate to war, or to the presence versus absence of troops at their permanent duty assignments. These studies have been restricted to time periods of less than a year [5] or a population of deployed personnel only [6]. The present study was conducted to assess Ct incidence and recurrent infection rates in a cohort of active component Army personnel, and determine any association of these rates with soldier transience, in addition to demographic characteristics and STI history.

## Methods

### Study location and population

The location chosen for this study was Fort Bragg, North Carolina (NC), one of the largest military installations in the country, with an active duty population numbering more than 50,000 [7] which is dominated by elite Army units and which consists of an exceptional range of occupational groups assigned there well reflecting the Army as a whole. The population at this military base and the period chosen likely provided valid data to explore the effects of troop mobility on Ct transmission at or near a long-term duty location (“permanent duty station”; assignments usually lasting 3 years or more).

We retrospectively followed a cohort of active duty Army soldiers for Ct diagnoses. Any soldier with a duty assignment at Fort Bragg, NC between January 1, 2005 and June 14, 2010 was eligible for entry into the cohort. Although ready access to sensitive diagnostic testing itself does not vary substantially among the major military bases [8], Fort Bragg does have a discretely named, centralized clinic to treat sexually transmitted infections—a resource not copied at all military installations—and generates a consistently high number of patient encounters for STIs. This likely increases the odds of both diagnostic testing and compliance with passive surveillance procedures.

### Case definition

Ct infections were ascertained from reportable medical event records generated from Fort Bragg. An incident infection was defined as an individual soldier’s first Ct diagnosis after entry into the cohort. A recurrent Ct infection was defined as the *first* Ct diagnosis 30 days or more after an incident Ct diagnosis during the study period. Ct diagnoses less than 30 days apart or incident Ct diagnoses on the same day as entry into the study were omitted from analysis. A 30-day time interval was used to avoid including diagnoses possibly due to persistent infections given nucleic acid-based tests have been reported to detect non-viable organisms up to 3 weeks post-treatment [9]. The term “recurrent” is used to avoid implying that reinfections were clearly distinguishable from persistent infections; though few if any treatment failures would be expected given that the STI clinic at Fort Bragg uses a reliable protocol.

### Data sources

Reportable medical events (RMEs) were obtained from the surveillance case registry maintained by the Armed Forces Health Surveillance Center (AFHSC) [10]. Preventive medicine personnel and individual clinics at military treatment facilities inform the AFHSC of reportable conditions weekly using an electronic reporting system, for which the necessary diagnostic criterion to establish Ct infection is either demonstration of *C. trachomatis* in a clinical specimen by detection of antigen or nucleic acid, or isolation of the organism by culture. Ct and *Neisseria gonorrhoeae* infections at Fort Bragg are diagnosed through direct DNA detection (GenProbe, San Diego, California, U.S.), using urine specimens from male and female patients, or using swab specimens (cervical or vaginal) from female patients. Whenever a specimen produces a Ct-positive result, it is reported as a case of Ct infection through both military and civilian channels, the patient is treated with single-dose azithromycin (or a doxycycline regimen for macrolide-sensitive individuals), and scheduled for a clinical follow-up visit approximately one month after the initial encounter. During the study period, clinic personnel were routinely performing a test of cure for Chlamydia clearance at this early follow-up encounter. The database used for this study did not distinguish between cases discovered during routine screening and those detected when patients sought care for STI-related symptoms and signs.

Reportable disease and patient encounter data from the U.S. Military Health System are linkable to administrative and occupational data, including changes in duty assignment and significant periods of time away from a given base or installation where troops are assigned—specifically for deployment in support of a military operation. The Defense Medical Surveillance System (DMSS) [11], a database which captures medical and service-related data throughout an individual’s service in the military, was used to obtain sociodemographic and service-related covariates for cohort members—including age, race, sex, pay grade, highest education attained, marital status, history of gonococcal or chlamydial infection, prior duty assignments, prior deployments, deployments during the study, and other, cumulative time absent from Fort Bragg during the study. Coinfection data represented actual disease reports while prior infection data relied on recorded patient histories.

### Statistical analysis

Crude incidence rates of incident and recurrent infection were calculated. Soldiers were passively followed until occurrence of a Ct event (incident or first recurrent infection) or a censoring event. For the incident Ct cohort, follow-up time was calculated as the interval from the start of assignment at Fort Bragg during the study period to either time of incident Ct diagnosis or censoring. For the recurrent Ct cohort, the interval was calculated from 30 days after an incident Ct diagnosis to either the time of first recurrent infection or censoring. For both incident and recurrent Ct analysis, censoring events were transfer to another installation (permanent change in station), or separation from the Army including retirement, or the end of the study. Soldiers with no record of recurrent infection were assumed free of subsequent infection during the study period. For each soldier, any person-time associated with deployments or gaps in duty assignment at Fort Bragg was excluded from incidence rate calculations.

Sociodemographic and service-related characteristics were described for both incident and recurrent Ct infection cohorts. To compare whether soldiers who were excluded were different from those members in the relevant cohort, we used Pearson Chi-square and Kruskal-Wallis tests of significance for categorical and continuous variables, respectively. We used a Cox proportional hazards model to assess the association of characteristics at study entry (assignment at Fort Bragg) with time to incident Ct infection; time-varying characteristics were remeasured prior to entry into the recurrent Ct cohort (i.e. at time of incident Ct infection) to assess their association with time to first recurrence. The log rank test statistic and Kaplan-Meier survival curves were used to determine whether survival times were similar for each characteristic. Characteristics associated with time to incident Ct infection in univariate analysis (p < 0.10) and that met the proportional hazards assumption (p < 0.05) were assessed and adjusted for in multivariate analysis. Similar covariate analyses were conducted to assess association with time to recurrent Ct infection. Covariates eligible for adjustment and having contiguous risk were collapsed into categories for multivariate analysis (e.g. race-ethnicity and months in service at entry). Marital status was grouped into two categories: single and non-single (married and other) statuses. Age was grouped into three categories based on a tertile distribution. All univariate and multivariate analyses were stratified for gender. Data management and statistical analyses were conducted using Statistical Analysis Software (SAS version 9.2, Cary, North Carolina, U.S).

The Division of Human Subjects Protection at the Walter Reed Army Institute of Research (WRAIR) reviewed the protocol and deemed the project (WRAIR #1750), which used pre-existing data that were not linked to an individual’s identifiers, exempt from human subjects review by the WRAIR Institutional Review Board.

## Results

The final cohort of 67,425 soldiers excluded 113 persons who were deployed throughout the study period, and 4 who were infected at the time they entered into the cohort at the start of the study (initially prevalent cases). The cohort was predominantly male (87%), and included ages ranging from 17 to 67 years (median 24.0 years, mean 25.8 years). Sixty-five percent had a rank below sergeant (i.e., had one of the 4 lowest military pay grades, E1 through E4). The relative proportions of males and females were similar in the excluded and cohort populations, but the excluded soldiers were significantly older (median 32.0 years, mean 31.5 years) and of higher pay grade (82% E5 and above, p < 0.0001).

### Incident infections

Among 67,425 soldiers assigned to Fort Bragg during January 1, 2005 to June 14, 2010, 2,198 individuals (3.3%) were reported to have had at least one Ct infection. The total number of both initial and recurrent infections (n = 223), excluding 72 that occurred within 30 days of a prior Ct diagnosis, was 2,493. The crude incidence rate was 21.7 per 1,000 person-years of follow-up (95% confidence interval (CI), 20.8 to 22.6), excluding recurrent cases. The median time to an initial infection was 8.8 months (interquartile range (IQR), 3.9 to 19.3) after entry. Time to an initial infection was shorter in females (median 6.4 months, IQR 3.1 to 14.7 months) than males (median 11.1 months, IQR 4.7 to 22.2 months). The follow-up period for the cohort ranged from 1.8 months to 5.4 years (median 1.4 years, IQR 0.7 to 2.1 years); males were followed for slightly longer (median 1.4 years, IQR 0.8 to 2.1 years) compared to females (median 1.2 years, IQR 0.6 to 2 years). Among 694 initially infected personnel who had deployed for a combat mission, 50% received their Ct diagnosis within 6 months of return to Fort Bragg, while a quarter of them did not receive a Ct diagnosis for at least a year after their return. In 101,149 person-years of follow-up (Table 1), the crude incidence rate of incident Ct per 1,000 person-years was substantially higher among females (73.2, 95% CI, 68.6 to 78.1) than among males (14.4; 95% CI, 13.6 to 15.2); an overall infection rate five times higher in women (unadjusted hazard ratio (HR) 5.0, 95% CI 4.6 to 5.5) than in men.

**Table 1:** **Univariate analysis of sociodemographic and service-related characteristics associated with incident *Chlamydia trachomatis *** **infection among female and male soldiers, Fort Bragg, 2005-2010**

			**Female**						**Male**			
	**TOTAL (n = 8909)**	**PY (total = 12586.80)**		**Infected (total n = 922) n (%)**	**Hazard ratio**	**95% CI**	**TOTAL (n = 58516)**	**PY (total = 88563.06)**		**Infected (total n = 1276) n (%)**	**Hazard ratio**	**95% CI**
Age (years)												
17-20	2060	2694.47	426	(46)	6.60	(5.40-8.06)	14042	20964.21	468	(37)	3.71	(3.15-4.35)
21-25	3273	4494.38	372	(40)	3.49	(2.85-4.28)	21334	30932.45	592	(46)	3.18	(2.72-3.72)
26-67	3576	5397.95	124	(14)	1.00		23139	36663.82	216	(17)	1.00	
Missing	0	0.00	0				1	2.58	1			
Race/ethnicity												
Non-white	4667	6659.46	568	(62)	1.42	(1.24-1.62)	17595	26364.53	682	(54)	2.66	(2.38-2.97)
Black	3093	4396.98	380	(42)	1.44	(1.24-1.66)	8913	13172.93	477	(38)	3.72	(3.30-4.20)
Hispanic	1015	1493.41	121	(13)	1.36	(1.11-1.67)	6030	9293.29	157	(12)	1.74	(1.46-2.08)
Other	559	769.07	67	(7)	1.44	(1.11-1.87)	2652	3898.31	48	(4)	1.26	(0.94-1.69)
White	4048	5622.83	343	(38)	1.00		39916	60480.45	587	(46)	1.00	
Unknown	194	304.51	11				1005	1718.09	7			
Pay grade												
E1-E4	5674	7555.88	832	(90)	5.87	(4.72-7.30)	38439	55897.88	1101	(86)	3.59	(3.06-4.21)
E5+	3235	5030.92	90	(10)	1.00		20077	32665.18	175	(14)	1.00	
Education												
High school or less	6270	8561.25	823	(91)	4.27	(3.40-5.35)	43876	65941.18	1171	(94)	4.52	(3.60-5.69)
Some college or higher	2477	3770.11	82	(9)	1.00		13069	19997.24	78	(6)	1.00	
Unknown	162	255.44	17				1571	2624.65	27			
Marital status												
Single	4743	6443.52	667	(72)	2.43	(2.10-2.80)	34258	50574.89	936	(73)	2.03	(1.79-2.30)
Non-single	4156	6122.33	254	(28)	1.00		24219	37913.03	340	(27)	1.00	
Unknown	10	20.95	1				39	75.14	0			
History of prior PCS												
0-3	5351	7280.65	739	(80)	2.87	(2.44-3.37)	36731	54302.64	968	(76)	1.95	(1.71-2.21)
4-34	3558	5306.15	183	(20)	1.00		21785	34260.42	308	(24)	1.00	
Breaks in duty at Ft. Bragg												
Yes	712	1306.37	894	(3)	0.29	(0.20-0.42)	3941	7118.61	30	(2)	0.28	(0.19-0.40)
No	8197	11280.42	28	(97)	1.00		54575	81444.45	1246	(98)	1.00	
History of prior deployments												
1-3	3338	5726.58	33	(4)	0.31	(0.22-0.44)	30292	54647.42	133	(10)	0.75	(0.63-0.90)
None	5571	6860.22	889	(96)	1.00		28224	33915.64	1143	(90)	1.00	
Deployment during study												
Yes	3338	5726.58	149	(16)	0.25	(0.21-0.29)	30292	54647.42	402	(32)	0.29	(0.26-0.33)
No	5571	6860.22	773	(84)	1.00		28224	33915.64	874	(68)	1.00	
History of Ct												
Yes	959	1299.81	98	(11)	1.02	(0.83-1.26)	1186	1706.80	48	(4)	2.00	(1.50-2.66)
No	7950	11286.99	824	(89)	1.00		57330	86856.27	1228	(96)	1.00	
History of GC before entry and initial Ct												
Yes	163	209.38	25	(3)	1.48	(0.99-2.20)	408	632.66	49	(4)	5.29	(3.98-7.04)
No	8746	12377.42	897	(97)	1.00		58108	87930.40	1227	(96)	1.00	
Months in service at entry												
0 to 6	2081	2896.03	359	(39)	2.99	(2.56-3.49)	16900	25143.42	434	(34)	1.65	(1.45-1.88)
7 to 14	2028	2660.21	274	(30)	2.42	(2.05-2.85)	13010	19074.37	383	(30)	1.90	(1.66-2.18)
15 to 297	4800	7030.56	289	(31)	1.00		28606	44345.26	459	(36)	1.00	
Primary occupation specialty at entry												
Combat	1782	2673.2	110	(12)	0.51	(0.42-0.62)	21004	32014.6	339	(27)	0.64	(0.57-0.73)
Other	7127	9913.60	812	(88)	1.00		37512	56548.47	937	(73)	1.00	

Note: CI - confidence interval; Other marital status - married, other; Other race/ethnicity - other, Asian/Pacific Islander, American Indian/Alaskan native; PCS - permanent change in station.

Significantly lower rates of infection were found among women (adjusted hazards ratio (AHR) 0.24; 95% CI, 0.20 to 0.28) and men (AHR 0.28; 95% CI, 0.25 to 0.32) who deployed for a military operation during the period of study, and among women (AHR 0.31; 95% CI, 0.21 to 0.45) and men (AHR 0.28; 95% CI, 0.20 to 0.41) who had breaks in duty from the garrison setting for an extended period due to reasons other than deployment (Table 2). Lower rates were also noted among female cohort members with a history of prior deployment for a wartime mission (AHR 0.58; 95% CI, 0.40 to 0.85), or whose occupation was combat-related (AHR 0.73; 95% CI, 0.60 to 0.90) (Table 2). Younger age, lower pay grade, less education, being single, and non-white race-ethnicity designation were all associated with higher Ct infection rates among both female and male cohort members (Tables 1 and 2). A history of GC infection prior to Ct infection was an additional risk factor in men.

**Table 2:** **Multivariate analysis of sociodemographic and service-related characteristics associated with incident *Chlamydia trachomatis *** **infection among female and male soldiers, Fort Bragg, 2005-2010**

		**Female**		**Male**
	**Hazard ratio**	**95% CI**	**Hazard ratio**	**95% CI**
Age (years)				
17-20	2.64	(2.03-3.43)	2.24*	(1.85-2.71)
21-25	1.94	(1.53-2.46)		
26-67	1.00		1.00	
Missing				
Race/ethnicity				
Non-white	1.48	(1.29-1.70)	-	-
Black	-	-	3.79	(3.33-4.31)
Hispanic	-	-	1.62^	(1.38-1.89)
Other	-	-		
White	1.00		1.00	
Unknown				
Grade				
E1-E4	2.21	(1.65-2.97)	1.99	(1.60-2.48)
E5+	1.00		1.00	
Education				
High school or less	1.54	(1.18-2.00)	2.22	(1.75-2.83)
Some college or higher	1.00		1.00	
Unknown				
Marital status				
Single	1.34	(1.14-1.58)	1.19	(1.03-1.38)
Non-single	1.00		1.00	
Unknown				
History of prior PCS				
0-3	1.09	(0.87-1.36)	1.04	(0.87-1.24)
4-34	1.00		1.00	
Breaks in duty at Ft. Bragg				
Yes	0.31	(0.21-0.45)	0.28	(0.20-0.41)
No	1.00		1.00	
History of prior deployments				
1-3	0.58	(0.40-0.85)	1.08	(0.88-1.33)
None	1.00		1.00	
Deployment during study				
Yes	0.24	(0.20-0.28)	0.28	(0.25-0.32)
No	1.00		1.00	
History of Ct	NS			
Yes	-	-	1.34	(0.97-1.86)
No	-	-	1.00	
History of GC before entry and initial Ct	NS			
Yes	-	-	3.60	(2.63-4.93)
No	-	-	1.00	
Months in service at entry				
0 to 14	1.13	(0.93-1.37)	0.96	(0.81-1.13)
15 to 297	1.00		1.00	
Primary occupation specialty at entry				
Combat	0.73	(0.60-0.90)	0.93	(0.82-1.06)
Other	1.00		1.00	

Note: CI - confidence interval; NS - not significant; PCS - permanent change in station.

*Age groups 17–20 and 21–25 were collapsed into a single group.

^Race/ethnic groups Hispanic and Other were collapsed into a single group.

### Recurrent infections

Ninety-four of the 2,198 personnel who had experienced an initial infection were lost to further follow-up because they were deployed until either the end of their assignment or the end of the study. Although those lost to follow-up were similar to cohort members in age, race, marital status, and education attained at the start of the recurrent infection study, they were significantly different in grade (97% excluded the lowest 4 military pay grades, E1 through E4, compared to 87% of the cohort, p = 0.007). Among the remaining 2,104 personnel who had an incident Ct infection, 223 (10.6%) experienced a recurrent infection at least once during a median of 9.7 months of follow-up (IQR,4.1 to 16.9). During follow-up, 31 (13.9%) of the 223 with an initial recurrent infection had a second recurrent diagnosis of Ct and 3 (1.3%) had a third. The crude incident recurrence infection rate among soldiers previously infected during the study period was 110.7 per 1,000 person-years of follow-up (95% CI, 96.9 to 126.0). Females had a higher crude incidence rate (161.9 per 1,000 person-years, 95% CI, 136.8 to 190.53; compared to male cohort members who had an incidence rate of 72.2 per 1,000 person-years (95% CI, 57.9 to 89.1), translating to more than twice the rate (HR 2.3, 95% CI 1.7 to 3.0) of that in men. Recurrent infection occurred within a median 5.8 months (IQR, 2.4 to 15.5). Male cases had shorter time to recurrent Ct infection after entry (median, 3.9 months, IQR 2.1 to 12.6 months) compared to female cases (median, 6.7 months, IQR 2.7 to 15.9 months).

After controlling for other significant covariates (see Table 3 for unadjusted hazard ratios), higher rates of recurrent infection in female members were among those in the youngest group (through age 20, AHR 2.39, 95% CI, 1.12 to 5.12), and those without any post-high school education (AHR, 3.59; 95% CI, 1.12 to 11.52) (Table 4). Age and education were not significant factors for recurrent infection in males (Table 4). A history of any prior overseas deployment (AHR, 0.28; 95% CI, 0.13 to 0.62) was associated with a lower recurrent infection rate in women (Table 4). However, among those with a recurrent infection and a history of deployment (n = 79), 46% were diagnosed with recurrent Ct within 6 months post-deployment.

**Table 3:** **Univariate analysis of sociodemographic and service-related characteristics associated with recurrent *Chlamydia trachomatis *** **infection among female and male soldiers, Fort Bragg, 2005-2010**

			**Female**						**Male**			
	**Total (n = 890)**	**PY (total = 864.25)**		**Reinfected (total n = 140) n (%)**	**Hazard ratio**	**95% CI**	**Total (n = 1214)**	**PY (total = 1149.05)**		**Reinfected (total n = 83) n (%)**	**Hazard ratio**	**95% CI**
Age (years)												
17-20	281	282.50	65	(46)	3.33	(1.76-6.31)	211	224.28	25	(30)	2.42	( 1.26-4.65)
21-25	453	425.74	64	(46)	2.13	(1.12-4.05)	710	622.89	44	(53)	1.43	(0.78-2.61)
26-67	156	156.02	11	(8)	1.00		293	301.88	14	(17)	1.00	
Race/ethnicity												
Non-white	554	544.90	96	(68)	1.27	(0.89-1.82)	649	618.79	54	(66)	1.62	(1.03-2.56)
Black	374	359.95	66	(47)	1.31	(0.89-1.92)	454	419.25	41	(50)	1.79	(1.10-2.89)
Hispanic	116	115.33	20	(14)	1.26	(0.74-2.14)	148	151.29	12	(15)	1.53	(0.78-3.01)
Other	64	69.62	10	(7)	1.08	(0.54-2.14)	47	48.25	1	(1)	0.41	(0.05-2.98)
White	325	310.78	44	(31)	1.00		558	520.43	28	(34)	1.00	
Unknown	11	8.58	0	(0)			7	9.82	1			
Grade												
E1-E4	800	754.99	135	(96)	3.72	(1.52-9.08)	1042	959.94	70	(84)	0.99	(0.54-1.78)
E5+	90	109.27	5	(4)	1.00		172	189.11	13	(16)	1.00	
Education												
High school or less	794	758.43	132	(84)	5.13	(1.63-16.12)	1111	1047.01	74	(74)	0.94	(0.41-2.16)
Some college or higher	81	90.61	3	(16)	1.00		76	84.11	6	(26)	1.00	
Unknown	15	15.22	5				27	17.93	3			
Marital status												
Single	647	611.94	104	(82)	1.18	(0.80-1.72)	885	799.72	68	(98)	1.87	(1.07-3.27)
Non-single	242	251.09	36	(18)	1.00		329	349.33	15	(2)	1.00	
History of prior PCS												
0-3	709	689.73	121	(86)	1.63	(1.04-2.64)	914	852.45	65	(78)	1.23	(0.73-2.07)
>3	181	174.53	19	(14)	1.00		300	296.60	18	(22)	1.00	
Breaks in duty at Ft. Bragg												
Yes	46	62.23	3	(2)	0.29	(0.09-0.92)	59	85.79	2	(2)	0.35	(0.09-1.43)
No	844	802.03	137	(98)	1.00		1155	1063.26	81	(98)	1.00	
History of prior deployments												
1-3	168	144.94	9	(6)	0.33	(0.17-0.65)	478	379.33	30	(64)	1.04	(0.66-1.63)
None	722	719.32	131	(94)	1.00		736	769.72	53	(36)	1.00	
GC diagnosis at initial Ct infection												
Yes	49	45.11	10	(7)	1.38	(0.73-2.64)	91	79.11	8	(10)	1.38	(0.66-2.86)
No	841	819.15	130	(93)	1.00		1123	1069.93	75	(90)	1.00	
Months in service at entry												
0 to 24	541	560.15	103	(74)	1.59	(1.09-2.32)	510	530.55	46	(55)	1.54	(1.00-2.37)
25 to 292	349	304.11	37	(26)	1.00		704	618.50	37	(45)	1.00	
Primary occupation specialty at entry												
Combat	106	110.07	9	(6)	0.49	(0.25-0.96)	317	299.18	24	(29)	1.16	(0.72-1.86)
Other	784	754.19	131	(94)	1.00		897	849.87	59	(71)	1.00	

Note: CI - confidence interval; Other marital status - married, other; Other race/ethnicity - other, Asian/Pacific Islander, American Indian/Alaskan native; PCS - permanent change in station.

**Table 4:** **Multivariate analysis of sociodemographic and service-related characteristics associated with recurrent *Chlamydia trachomatis *** **infection among female and male soldiers, Fort Bragg, 2005-2010**

	**Female**		**Male**	
	**Hazard ratio**	**95% CI**	**Hazard ratio**	**95% CI**
Age (years)				
17-20	2.39	(1.12-5.12)	1.70	(0.78-3.72)
21-25	1.83	(0.88-3.80)	1.13	(0.59-2.16)
26-67	1.00		1.00	
Race/ethnicity	NS			
Non-white	-	-	1.68	(1.06-2.65)
White	-	-	1.00	
Grade			NS	
E1-E4	1.62	(0.58-4.45)	-	-
E5+	1.00		-	-
Education			NS	
High school or less	3.59	(1.12-11.52)	-	-
Some college or higher	1.00		-	-
Marital status	NS			
Single	-	-	1.61	(0.88-2.94)
Non-single	-	-	1.00	
History of prior PCS			NS	
0-3	1.06	(0.58-1.92)	-	-
>3	1.00		-	-
Breaks in duty at Ft. Bragg				
Yes	0.32	(0.10-1.02)	0.37	(0.09-1.51)
No	1.00		1.00	
History of prior deployments			NS	
1-3	0.28	(0.13-0.62)	-	-
None	1.00		-	-
GC diagnosis at initial Ct infection	NS		NS	
Yes	-	-	-	-
No	-	-	-	-
Months in service at entry				
0 to 24	0.84	(0.51-1.39)	1.18	(0.71-1.96)
25 to 292	1.00		1.00	
Primary occupation specialty at entry			NS	
Combat	0.59	(0.30-1.16)	-	-
Other	1.00		-	-

Note: CI - confidence interval; NS - not significant; PCS - permanent change in station.

## Discussion

In this retrospective cohort study conducted among active duty soldiers stationed at Fort Bragg, NC, the crude incidence rate of Ct infection of 21.7 per 1,000 person-was nearly twice the rate (11.4 per 1,000 person-years) previously reported for active duty military personnel by the Armed Forces Health Surveillance Center during 2008 to 2009 [12], which raises a suspicion about the influence of high STI rates in the southeastern United States. National notifiable disease data indicate that the crude incidence rate of Ct per 1,000 population ranged from 3.02 in the New England area to 5.10 per 1,000 in the East South Central region of the U.S., though it should be noted that figures are not age- or sex-adjusted for direct comparison to military populations [13]. In their Ct prevalence study of female Army recruits, Gaydos and colleagues found an association with origin from any of 5 southern states, including East (Alabama, Mississippi) and West (Louisiana) South Central states. The implicated South Atlantic states (South Carolina, Georgia) did not include North Carolina [14]. However, state-level data show that during 2005 to 2009, the average annual incidence in Cumberland County—which surrounds Fort Bragg—was 7.99, and this was twice the rate for North Carolina as a whole during the same period (3.91) [15].

About 1 in 10 Fort Bragg soldiers who were found to have an incident Ct infection in this study were later discovered to have Ct again, on average within a year of the initial infection. There were 223 new positive Ct diagnoses overall, translating to a crude recurrent infection rate of 110.7 per 1,000 person-years of follow-up. This rate is generally in line with other studies, particularly if accounting for differences in population selectivity [16,17]. Since some proportion of presumed incident infections at Fort Bragg likely represents prevalent infections that had not been treated, but were detected on screening, the rate of recurrent infection may serve as a better indicator of locally endemic disease, or of the behavioral and other risk factors influencing transmission.

Our analysis supports previously published findings regarding the association of Ct infection with young age [18], [12,14,16,17,19-25] black race [14,16,18,20-23,26,27], not having more than a high school education [23] and being single [18]. Young age was not found to be associated with recurrent infection in men, and this supports the findings of Dunne and colleagues. [28] In that study, however, men with less than a high school education appeared to be at greater risk of recurrent infection, whereas at Fort Bragg the lower recurrent infection rate associated with a higher education level was only found to hold in women after adjustment for significant covariates.

Overall, women were more than twice as likely to experience recurrent infection as men. Since directly observed, single-dose azithromycin treatment is a reliable procedure at Fort Bragg—with only rare cases of macrolide sensitivity prompting a doxycycline regimen instead—few if any of the presumed reinfections were likely to represent non-compliance, treatment failure, relapse, or persistent infection. The likelihood of screening despite no genitourinary symptoms was probably greater among women; and this may also underlie the significant difference in the time elapsed between initial and subsequent infection, which was a median of 6.7 months for females, but only 3.9 months for males, in whom symptoms may have prompted follow-up screening more often than in women. Time away from Fort Bragg (deployment as well as other breaks in duty) was associated with a lower Ct infection rate in both men and women. Non-deployment breaks in duty were also analyzed with respect to recurrent Ct infection, and were associated with a lower recurrence rate among women (though the adjusted hazard ratio did not reach statistical significance). The mobility-related findings were adjusted for several factors including age, pay grade, marital status, race, and education. While opportunity bias may have affected the findings to a degree, person-time from absence periods was not part of incidence rate calculations, and there were actually more person years of observation at Fort Bragg among those who experienced deployment (60,374) than among those who did not (40,776). It also happens that the number of soldiers who deployed during the study period (33,630) was similar to the number who did not (33,795).

Soldiers’ history of prior deployments, a longer term transience indicator than deployment or other breaks in duty during the Fort Bragg assignment, was associated with lower incident and recurrent infection rates only in women. During the study period, medical readiness screening of women prior to overseas deployment included Chlamydia testing. Thus there was increased compliance with periodic screening—and treatment as indicated—in women who deployed. This likely resulted in a smaller proportion of women having chronic, asymptomatic infection if they previously deployed, compared to women who had no deployment history. Similarly, women who are screened for Chlamydia may be more likely to take precautions to prevent infection or reinfection; and the screening encounter alone may raise sufficient awareness even among uninfected patients. Further study would be needed to bear this out.

The ‘healthy worker effect’ may have a role in the lower infection rates associated with deployment. Factors that medically or administratively preclude soldiers from deploying to a combat theater may also be associated with a higher risk of Ct infection. On the other hand, after deployed soldiers—particularly males--return to their home stations following a combat mission, their risk-taking behavior that was recently projected on the battlefield may be diverted toward their social interactions, including those with local civilian populations. This is supported by the preliminary findings of a behavioral survey conducted at Fort Bragg [personal communication, Womack Army Medical Center, Fort Bragg]. Behavioral factors may explain the diagnosis of approximately half of incident and recurrent infections within six months of return from a deployment.

The findings of this investigation should be interpreted with caution due to a number of factors. Firstly, the incidence of Ct may have been underestimated using RMEs alone. In a comparison of Ct laboratory results from 2010 to 2012 to RMEs, 36.1% (females) and 38.7% (males) who had a positive test result did not have a RME for Ct within 30 days of the result. However, the proportion of service members with RMEs and a positive test was >82% [29]. Secondly, Ct infection may occur or become manifest while soldiers are deployed, or during rest and relaxation periods away from the combat zone. Such cases would be treated before soldiers’ return to their home garrison setting, and would thus not be captured as disease reports that would permit a direct comparison to garrison rates.

Thirdly, compared to soldiers who attend the Fort Bragg STI clinic, those who avoid military facilities for Chlamydia treatment and self-pay or use alternative insurance to keep their infections from appearing in transferred health records may be very different with respect to one or more of the covariates examined in this study. Rates may thus be underestimated, and the effect of risk factors either diluted or exaggerated. On the other hand, the high Chlamydia rate at Fort Bragg compared to other military installations may be attributable not only to endemic disease in the local community, but also enhanced case finding and reporting at Fort Bragg compared to other military locations. With respect to possible underreporting across the military, the degree to which patients seek care outside of the military treatment facility is not known. Still, administrative and patient care factors probably do not account for most of the variance in Ct rates within the Army, as higher rates are noted at locations where civilian care is readily available.

Finally, valid comparisons between women and men are limited by differences in regular screening practices. By U.S. military policy women under 30 years of age are regularly screened for Ct during annual well woman examinations and military women younger than 26 years of age are routinely screened for Ct infection during their initial entry training. Also, distinguishing reinfection from persistent infection is difficult; and two additional, related factors are the high proportion of Ct-infected persons without symptoms, and variability in clearance of the infection. Lechner et al. determined prevalence rates of 15% and 11% for females and males, respectively, when they screened sexually active young adults, as well as adolescents who were children of military personnel. These were asymptomatic patients attending military-run clinics in San Antonio [30]. The relatively small difference in rates between asymptomatic females and males suggest that when male rates are calculated primarily from those seeking care for urethritis they may underestimate the incidence or prevalence of actual infections. Information regarding the reason for diagnostic testing, compliance with antimicrobial treatment, treatment of partners, and tests of cure could not be determined from the available data in the present study.

Despite its limitations, this descriptive study provides evidence of sufficient statistical power to influence ongoing surveillance and prevention efforts. The population observed for this study is substantially larger than sample populations in most other published reports about Ct risk factors and recurrent infection that use data from a single geographic location—with the notable exception of descriptive analyses based on national or multi-regional surveillance data [17,23]. Moreover, the cohort represents a broad cross-section of the U.S. population as every region contributes to the military population [31,32] and therefore may better permit generalizing results for comparison to national notifiable disease data, perhaps to a greater degree than other studies of predominantly young populations based on a specific state, city, college, or school district.

## Conclusions

This investigation revealed evidence suggesting that during a period of war many members of elite Army units faced a relatively high risk of contracting Ct at or near their permanent duty location; and that spending time away from that location—including combat duty time—was associated with a lower cumulative risk of infection during periods before and after those absences. While there may have been short periods of increased risk just prior to departures, or for a time after each return, the total risk was calculated to be lower for the more mobile soldiers. The findings support the STI prevention efforts at Fort Bragg and the surrounding community, regardless of how often or for how long soldiers have deployed for military operations. They also warrant more liberal use of diagnostic screening for Ct during scheduled follow-up visits that succeed all STI encounters. Specifically, the median interval to recurrent infection determined in this study directly supports the recommendations of the Centers for Disease Control and Prevention to repeat diagnostic testing at approximately 3 months after the initial diagnosis [33].

## Abbreviations

AFHSC: Armed Forces Health Surveillance Center; AHR: Adjusted hazards ratio; CI: Confidence interval; Ct: *Chlamydia trachomatis*; DMSS: Defense Medical Surveillance System; GC: Gonorrhea; IQR: Interquartile range; NC: North Carolina; RMEs: Reportable medical events; STI: Sexually transmitted infection.

## Competing interests

The authors declare that they have no competing interests relevant to the manuscript submitted to BMC Public Health.

## Authors’ contributions

SH and PS developed the protocol. BP and SH drafted the manuscript. SH, FL, and BP conducted statistical analysis of the data. SH, RO, KF, NM, PS, and BP interpreted the data and revised the manuscript. All authors read and approved the final manuscript.

## Authors’ information

U.S. Department of Defense Disclaimer:

The views expressed are those of the authors and should not be construed to represent the positions of the U.S. Department of Defense.

## Pre-publication history

The pre-publication history for this paper can be accessed here:

http://www.biomedcentral.com/1471-2458/14/181/prepub
